# Profiling of miR-205/P4HA3 Following Angiotensin II-Induced Atrial Fibrosis: Implications for Atrial Fibrillation

**DOI:** 10.3389/fcvm.2021.609300

**Published:** 2021-04-26

**Authors:** Zezhou Xiao, Desai Pavan Kumar Reddy, Chuqing Xue, Ximao Liu, Xiong Chen, Jiale Li, Xiao Ling, Shaoyi Zheng

**Affiliations:** Department of Cardiovascular Surgery, Nanfang Hospital, Southern Medical University, Guangzhou, China

**Keywords:** MiR-205, P4HA3, atrial fibrosis, atrial fibroblast, atrial fibrillation, proliferation, migration, JNK signaling pathway

## Abstract

**Objective:** Atrial fibroblasts are the main component of atrial fibrosis. Data in previous studies proved the implication of miRNAs in AF progression and the association of miR-205 with cancer associated-fibroblasts, while no evidence supported the implication of miR-205 in atrial fibrosis. Therefore, this study aims to explore the effect and mechanism of miR-205/P4HA3 axis on atrial fibrosis.

**Methods:** Angiotensin II (Ang II) was used to induce atrial fibrosis model in rats, which was verified by H&E staining and Masson staining. qRT-PCR and Western blot were applied to measure the expressions of miR-205, P4HA3, collagen I, and α-SMA. The rat atrial fibroblasts were isolated and then subjected to Ang II treatment or cell transfection for determination of cell biological functions using CCK-8, BrdU assay, TUNEL staining, and cell scratch assay. qRT-PCR and Western blot was applied to analyze the expressions of miR-205, P4HA3, collagen I, α-SMA, JNK, and p-JNK in atrial fibroblasts. Dual-luciferase reporter gene assay and RNA immune-precipitation experiment was employed to verify the binding relationship between miR-205 and P4HA3.

**Results:** Ang II induced rats had disordered arrangement of atrial muscles with uneven nuclear sizes and necrotic atrial myocytes, and increased collagen deposition, in which elevated expressions of P4HA3, collagen I, and α-SMA as well as suppressed expression level of miR-205 were found. *In vitro*, Ang II treatment in atrial fibroblasts with overexpression of P4HA3 facilitated cellular migration and proliferation, with the induction of JNK signaling pathway. However, these trends were reversed after transfection with miR-205 mimic. P4HA3 is a target gene of miR-205.

**Conclusion:** The miR-205/P4HA3 axis is implicated in atrial fibrosis by inhibition of rat fibroblast proliferation and migration and the inactivation of JNK signaling pathway.

## Introduction

Atrial fibrillation (AF) has emerged as a growing concern in recent years as it continues to be the most commonly encountered arrhythmia in clinical practice (1, 2). However, currently available therapies bear considerable limitations, which consequently lead to incomplete efficacy, adverse effect risks, and a significant long-term recurrence rate (3). Atrial fibrosis is regarded as the primary substrate that maintains AF, and it is featured by excessive deposition of the extracellular matrix and abnormal proliferation of atrial fibroblasts (4, 5). These findings emphasize the relationship between AF and atrial fibrosis, although revealing the causal influence of tissue fibrosis on the occurrence and persistence of AF remains a crucial challenge. In addition, a better elucidation of the underlying mechanisms of atrial fibrosis is essential for identifying improved strategies for the treatment of structural remodeling. The renin-angiotensin system (RAS) is documented to be implicated in the course of AF (6). Serving as the major effector hormone of the RAS, Angiotensin II (Ang II) confers a crucial effect on the mediation of atrial fibrosis during AF (7). The rise in the Ang-II level interferes with myocardial hypertrophy, fibrosis, persistent hypertension, and adverse remodeling, which will trigger heart failure if left untreated (8). Herein, atrial fibrosis model was established by Ang-II infusion to unearth the specific mechanism in the settings of clinical AF.

MicroRNAs (miRNAs) are a broad range of small non-coding RNAs comprised of 21~25 ribonucleotides, which govern the expressions of complementary target messenger RNAs (9). Prior study has broadcasted that miRNAs is responsible for a variety of cardiovascular pathologies, including fibrosis, arrhythmia, ischemia, hypertrophy, atherosclerosis, and heart failure (10). Zhang et al. stated that miR-206 fortifies reactive oxygen specie production by targeting SOD1 in response to intrinsic cardiac autonomic nerve remodeling in AF (11). Furthermore, miR-21 has been expounded to confer inhibitory effect on cardiac fibroblast proliferation through upregulation of WWP-1 and inactivation of the TGF-β1/Smad2 signaling (12). As a less studied miRNA, miR-205 has elicited a crucial role in regulating cell migration and growth in cancers (13, 14). Although the studies referenced above have provided promising evidence in support of the performance of miRNAs in AF, whether miR-205 is dysregulated during AF is not clear. Prolyl 4-hydroxylase (P4H) is an evolutionarily conserved enzyme that is fundamental for the maintenance of collagen stability (15). Although it is beyond doubt that dysregulated P4HA is associated with a series of pathological processes, including tumor initiation and progression (16), there is no document supporting the implication of P4HA3 in AF. The JNKs are a class of stress-activated serine-threonine protein kinases of the mitogen-activated protein kinase (MAPK) (17). The development of cardiac diseases was reported to be related to the JNK signaling (18). Hence, the association between JNK signaling and the miR-205/P4HA3 axis in atrial fibrosis and the possible mechanism of their connection need to be analyzed from different perspectives.

We first compared in parallel the expression levels of miR-205/P4HA3 in Ang II induced atrial fibroblasts and atrial tissues. We then utilized the gain-of-function method, dual-luciferase reporter gene assay and RIP experiment to explore the interaction between miR-205 and P4HA3 and its implication in the pathogenesis of atrial fibrosis. Furthermore, we sought to determine whether the *in vitro* findings could be verified in rats with Ang II-induced atrial fibrosis, especially aiming at the impact of P4HA3 attenuation on atrial fibrosis and AF development. Finally, the relevant findings obtained from *in vitro* and animal models demonstrated that miR-205/P4HA3 axis is implicated in atrial fibrosis by inhibition of atrial fibroblast proliferation and migration and the JNK signaling pathway blockage.

## Materials and Methods

### Animals

Male Sprague Dawley (SD) rats (8 weeks, *n* = 12; 250 g) were randomly assigned to the Ang II group and Control group, and correspondingly given 0.15 mg/kg/day Ang II (Sigma-Aldrich, Merck KGaA, Darmstadt, Germany) and equal normal saline by intraperitoneal injection (19). Before injection, the Ang II had dissolved in 200 μL normal saline. Rats were euthanized after 3 weeks of continuous injection. The animal experimental procedures were approved by the Animal Research Committee of Center of Nanfang Hospital, Southern Medical University and were conducted with the Guide for the Care and Use of Laboratory Animals (National Institutes of Health Publication, 8th Edition, 2011).

### H and E Staining

The atrial tissues were collected from SD rats after the rats were anesthetized by intraperitoneal injection of 60 mg/kg pentobarbital sodium and 50 mg/kg ketamine. The atrial tissues were fixed in 4% paraformaldehyde for 24 h to make paraffin sections in 4 μm. Following deparaffinization by exposure to xylene, the paraffin sections orderly underwent gradient ethanol dehydration and 5 min of hematoxylin staining. After washing off the excess dye, the paraffin sections were subjected to 30 s of 1% hydrochloric alcohol (1 mlconcentrated hydrochloric acid + 99 ml 75% alcohol) differentiation and 2 min of eosin staining. Pictures were captured under a microscope after sections were given to dehydration, permeabilization, and seal.

### Masson Staining

Following deparaffinization and hydration, the paraffin sections were stained with Weigert's iron hematoxylin for 5 min, differentiated with hydrochloric alcohol and washed with tap water. The sections went through the following dyeing steps: 5 min of staining with ponceau-acid fuchsin solution, 3 min of treatment with phosphomolybdic acid solution, and 5 min of counterstain by toluidine blue-O. Sections were subjected to 1% glacial acetic acid for 1 min prior to dehydration, permeabilization, and seal. After that, each section was observed and photographed under a microscope.

### Isolation and Culture of Rat Atrial Fibroblasts

The two 14-day-old SD rats were anesthetized by intraperitoneal injection of pentobarbital sodium (60 mg/kg) and ketamine (50 mg/kg) before thoracotomy under sterile conditions. Then, the heart was isolated aseptically and placed in a petri dish. The atrial tissues were isolated from heart and washed to remove congestion and excess epicardial tissues. After that, atrial tissues were cut into pieces of about 1 mm^3^ in size followed by trypsin digestion and differential centrifugation. The atrial fibroblasts were obtained and infused in Dulbecco's modified Eagle medium (Gibco, Grand Island, NY, USA) with 10% fetal bovine serum for incubation at 37°C with 5% CO_2_. The culture medium was replaced every 3 day, and the cell morphology was observed and pictures were captured under an inverted microscope.

### Immunofluorescence

The atrial fibroblasts were identified by immunofluorescence analysis of Vimentin. Briefly, the atrial fibroblasts were fixed in 4% paraformaldehyde for 15 min and permeabilized with 0.5% Triton-X-100 for 20 min after being seeded in 24-well plate (5,000 cells/well). After that, sections were incubated with appropriate concentration of primary antibody against Vimentin (ab92547, 1:250, Abcam, Cambridge, MA, USA) overnight at 4°C, followed by exposure to the secondary antibody against Alexa Fluor 488-labeled goat anti-rabbit IgG (ab150077, 1:2000, Abcam, Cambridge, MA, USA) in the dark for 45 min. The secondary antibody was aspirated off, and sections were washed thrice with phosphate-buffered saline (PBS) for 5 min. Sections were subjected to 0.5 μg/mL DAPI prior to 5 min of incubation in the dark and 2 × 5 min of PBS washing. Before pictures were captured by fluorescence microscope (BX61FL, OLYMPUS, Tokyo, Japan), sections were given to the antifade mounting medium.

### qRT-PCR

The TRIzol (Invitrogen, Carlsbad, CA, USA) was utilized to extract total RNA. Reverse transcription was performed with the reverse transcription kit (TaKaRa, Tokyo, Japan). Gene expression was detected by employing a LightCycler 480 real-time PCR instrument (Roche, Indianapolis, IN, USA). The reaction conditions were consistent with the instructions of the fluorescent quantitative PCR kit (SYBR Green Mix, Roche Diagnostics, Indianapolis, IN). The thermal cycle parameter were 95°C for 10 s, followed by 45 cycles of 95°C for 5 s, 60°C for 10 s, and 72°C for 10 s. A final extension was presented at 72°C for 5 min. U6 or β-actin was used as an endogenous control for miRNA or mRNA normalization, and data analysis utilized 2^−ΔΔCt^ method. The test was done in triplicate. The formula is as follows: ΔΔCt = [Ct_(target gene)_−Ct_(reference gene)_]_experimental *group*_ −[Ct_(target gene)_−Ct_(reference gene)_] _control group_. The primer sequences are presented in Table 1.

**Table 1:** Primer sequence information.

**Name of primers**	**Sequences**
miR-205-F	ACACTCCAGCTGGGTGTCTGAGGCCACCTTA
miR-205-R	TGGTGTCGTGGAGTCG
U6-F	CTCTCGCTTCGGCAGCACA
U6-R	ACGCTTCACGAATTTGCGT
Collagen I-F	GCAATGCTGAATCGTCCCAC
Collagen I-R	CAGCACAGGCCCTCAAAAAC
α-SMA-F	GGCATCCACGAAACCACCTA
α-SMA-R	GTATGCGTGTGACGGCTCTA
P4HA3-F	GGGCTCAGCTATATGCCGTT
P4HA3-R	CAACTTTCCACGGGCAACTG
β-actin-F	TGAGCTGCGTTTTACACCCT
β-actin-R	GTTTGCTCCAACCAACTGCT

*F, forward primer; R, reversed primer*.

### Western Blot Analysis

The protein samples were acquired after cells were lysed with RIPA lysis buffer (Beyotime Biotechnology, Shanghai, China). The protein concentration was measured by a BCA kit (Beyotime), and the corresponding volume of protein (40 μg for each well) was added and mixed with loading buffer (Beyotime) in boiling-water bath for 3 min of denaturation. Electrophoresis was embarked for 30 min at 80 V and then for 1~2 h at 120 V once bromphenol blue reached the separation gel. After that, the proteins were transferred onto membranes at 300 mA for 60 min in ice-bath. Following rinsing 1~2 min with washing solution, the membranes were inactivated for 1 h at room temperature or sealed overnight at 4°C. The membranes were cultured with the primary antibodies against β-actin (ab8226, 1:1,000), Collagen I (ab34710, 1:1,000), α-SMA (ab32575, 1:1,000), P4HA3 (ab101657, 1:1,000), JNK (ab179461, 1:1,000), and p-JNK (ab124956, 1:1,000) (Abcam, Cambridge, MA, USA) at room temperature in a shaking table for 1 h. Before and after 1 h of incubation with the secondary antibody at room temperature, the membranes received 3 × 10 min of washing with washing solution. The chemiluminescence imaging analysis system (Gel Doc XR, Bio-rad) was applied for observation after membranes were given to developing liquid.

### Cell Transfection and Ang II Treatment

The rat atrial fibroblasts were subjected to transfection followed by exposure to Ang II for 24 h, and cells were assigned to following nine groups: the Control group (0 μM Ang II), Ang II-1 μM group (1 μM Ang II), Ang II-10 μM group (10 μM Ang II), miR-205 mimic+Ang II group (cotreatment with miR-205 mimic and 1 μM Ang II), mimic NC+Ang II group (simultaneous stimulation with mimic NC and 1 μM Ang II), pcDNA3.1-P4HA3+Ang II group (transfection with pcDNA3.1-P4HA3 prior to exposure to 1 μM Ang II), pcDNA3.1+Ang II group (transfection with pcDNA3.1 before treatment with 1 μM Ang II), miR-205 mimic+pcDNA3.1-P4HA3+Ang II group (cotransfection with miR-205 mimic and pcDNA3.1-P4HA3 followed by treatment with 1 μM Ang II) and SP600125 group [treatment with 20 μM JNK inhibitor SP600125 (Sigma-Aldrich, Saint Louis, MO, USA) for 24 h]. The miR-205 mimic, mimic NC (50 nM), pcDNA3.1-P4HA3, and pcDNA3.1 (20 μL) were supplied by GenePharma Co. Ltd (Shanghai, China). Transfection was embarked after usage of the Lipofectamine 2000 reagent (Invitrogen, Carlsbad, CA, USA).

### CCK-8 Assay

After transfection and Ang II treatment, the diluted suspension of atrial fibroblasts (1 × 10^6^ cells/mL) was seeded into a 96-well plate (100 μL/well). Three replicates were set for each sample. The plate was placed in an incubator for 0, 24, 48, 72, or 96 h, and then 10 μL CCK-8 kit (Tokyo, Dojindo, Japan) was added to each well of plate. After 2 h of additional incubation, the absorbance at 450 nm wavelength was measured.

### 5-Bromodeoxyuracil Nucleoside Assay

The fibroblasts were fixed in 4% paraformaldehyde for 20 min and washed twice with PBS. Then, slides received 2 N HCL (the ratio of PBS to HCL solution is 5:1) for 10 min and 0.1 M Borate buffer for 10 min at room temperature. Following PBS wash thrice, cells were inactivated in the blocking buffer for 60 min, incubated with the primary antibody against BrdU (ab6326, 1:250, Abcam, Cambridge, MA, USA) overnight at 4°C and washed three times with PBS. Cells were cultured with red fluorescence Alexa Fluor® 647-conjugated secondary antibody and washed thrice with PBS. Pictures were captured under a fluorescence microscope after DAPI staining of nuclei.

### TUNEL Staining

The slides were air-dried and fixed at room temperature for 30 min. Following PBS rinsing twice, slides were inactivated for 10 min. Before received the permeabilization solution of cell membrane at room temperature for 2 min, the slides were rinsed with PBS twice. Then, slides were reacted with a mixture of 2 μL TdT solution and 48 μL green fluorescent probe FITC-labeled dUTP solution (Beyotime, Shanghai, China) in a humidified and dark chamber for 1 h at room temperature. The slides were washed thrice with PBS and sealed with antifade mounting medium prior to observation under a fluorescence microscope. Cell nucleus was stained with DAPI. The number of TUNEL-positive cells (%) = number of red cells/total number of cells × 100%.

### Cell Scratch Assay

Cells in each group were seeded in six well-plates and cultured for 24 h at 37°C with 5% CO_2_ until the cell monolayer covered the bottom of the well. Cells in each group were inoculated in three wells. Following incubation, cells were scratched by using a 200 μL sterile pipette tip prior to PBS washing and 24 h of culture at 37°C with 5% CO_2_. Photographs were captured and scratch distance was recorded under a microscope at time 0 and 24 h post-cell scratch, and cell migration rate was calculated based on changes in scratch distance. The migration rate = (scratch distance at 0 h post-cell scratch - scratch distance at 24 h post-cell scratch) / scratch distance at 0 h post-cell scratch.

### Dual-Luciferase Reporter Gene Assay

The binding site of miR-205 and P4HA3 was predicted by the TargetScan (http://www.targetscan.org/vert_72/). The mutated type sequences and wild type sequences (mut-P4HA3 and wt-P4HA3) of the binding site were accordingly designed, synthesized and cloned into luciferase reporter gene vector pGL3-Promoter (Promega, Madison, WI, USA). Then, the vectors were cotransfected with miR-205 mimic or mimic NC (50 nM) into HEK293T cells. After cell transfection, Firefly luciferase activity and Renilla luciferase activity were determined by dual-luciferase reporter gene detection kit (Promega, Madison, WI, USA). Renilla luciferase activity was deemed as internal reference, and the relative activity was set as the ratio of firefly luciferase activity to Renilla luciferase activity.

### RNA Immunoprecipitation

Cells were washed with precool PBS for twice and centrifuged at 1,500 rpm for 5 min. Cell suspension was given RIP lysis buffer for cell lysis. The magnetic beads were resuspended in 100 μL RIP Wash Buffer, and the cells in the experimental group were subjected to 5 μg Argonaute-2 (Ago2) antibody (ab32381, 1:100, Abcam, Cambridge, MA, USA) and cells in the Control group were treated with IgG antibody for 30 min at room temperature. The centrifuge tube was placed on magnetic separation rack. The supernatant was discarded and tubes received 500 μL of RIP Wash Buffer for vortex oscillation, after which the supernatant was removed. The preceding process was repeated once prior to exposure to 500 μL RIP Wash Buffer for vortex oscillation and incubation on ice. The pretreated magnetic beads were placed on magnetic separation rack, the supernatant was removed and 900 μL RIP Immunoprecipitation Buffer was added into each tube. Cell lysates were centrifuged at 14,000 rpm at 4°C for 10 min, and 100 μL supernatant was aspirated into the magnetic bead-antibody complex for incubation overnight at 4°C. The complex was subjected to centrifugation and supernatant removal. Then, the centrifuge tube was washed with 500 μL RIP Wash Buffer and subjected to vortex oscillation before cell supernatant was abandoned. The sediments were washed six times. The samples were then incubated with Proteinase K Buffer (150 μL) to resuspend bead-antibody complexes at 55°C for 30 min, after which the tubes were placed on the magnetic separation rack to pipette off the supernatant. The expressions of miR-205 and P4HA3 were estimated by qRT-PCR after RNA extraction.

### Statistical Analysis

GraphPad prism7 software was applied to analyze data, and all data were exhibited as mean ± standard deviation (SD). *T*-test was employed to the comparison between two groups. Comparisons among multiple groups were assessed by one-way analysis of variance (ANOVA) and confirmed by Tukey's multiple comparisons test. *P* < 0.05 was regarded as statistically significant.

## Results

### Expressions of miR-205 and P4HA3 in Ang II-Induced Rat Atrial Fibrosis Model

**Figure 1:**
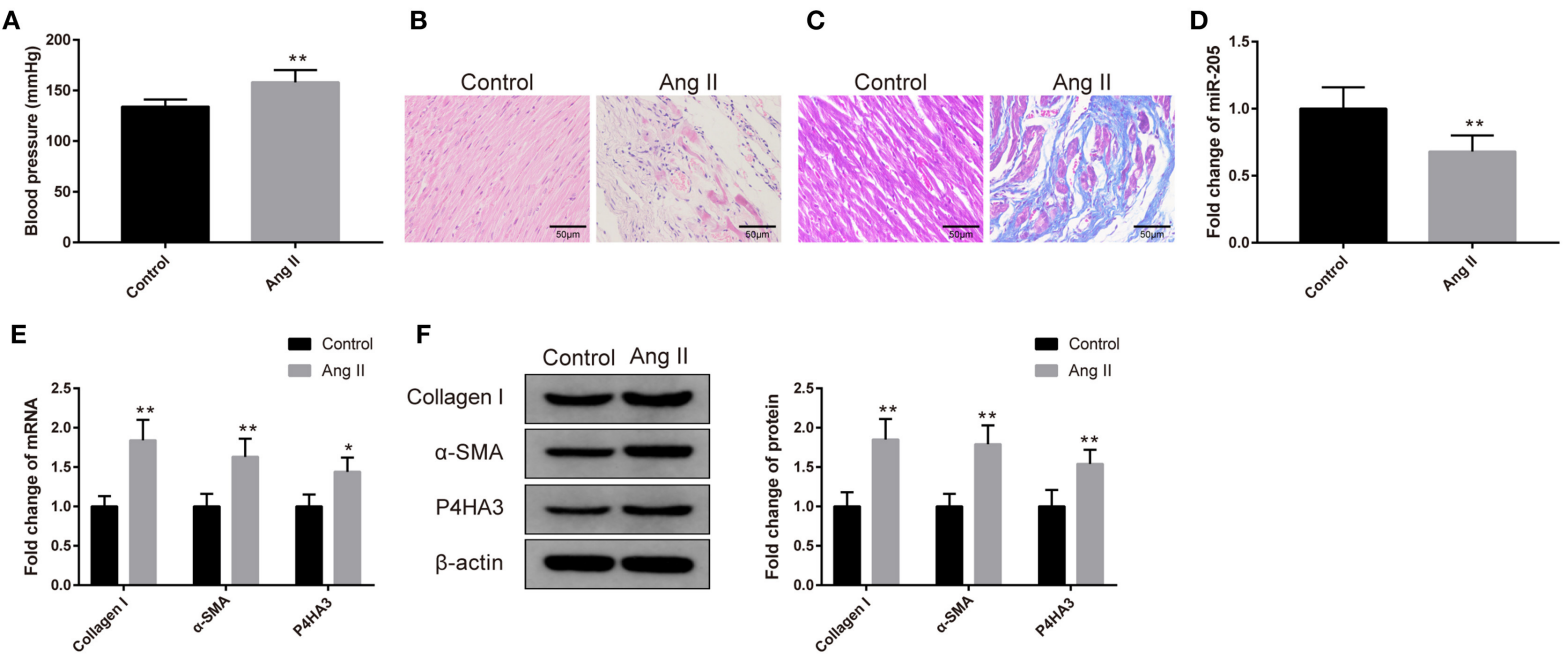
Rat atrial fibrosis model had elevated expression of miR-205 and decreased expression of P4HA3. The blood pressure of rats was measured **(A)** (*n* = 6). The pathology of rat atrial tissues was observed by H&E staining **(B)** (*n* = 6), and the collagen fiber deposition of rats was detected by Masson staining **(C)** (*n* = 6). qRT-PCR was utilized to inspect the mRNA level of miR-205 **(D)** (*n* = 6). The mRNA and protein levels of collagen I, α-SMA, and P4HA3 were analyzed by qRT-PCR **(E)** and Western blot **(F)** (*n* = 6); *P < 0.05, **P < 0.01.

Rats received 0.15 mg/kg Ang II by intraperitoneal injection had notable increased blood pressure (Figure 1A, *P* < 0.01). The atrial tissues were isolated prior to H&E and Masson staining. As depicted in Figure 1B, the atrial muscles in the Control group are neatly arranged and the nucleus is uniform, while the Ang II group has a disordered arrangement of atrial muscle with uneven nuclear sizes, and some atrial myocytes are necrotic. After Masson staining, the deposition of a small number of collagen fibers was found in the atrial muscle interstitium of the Control group, while a large number of blue cord-like areas were seen in the atrial muscle of the Ang II group. Compared with the Control group, the collagen fiber deposition of rats in the Ang II group was more notable (Figure 1C).

The qRT-PCR result yielded that the expression of miR-205 in the atrial tissues of the Ang II group was markedly reduced (Figure 1D, *P* < 0.01). Results of qRT-PCR and Western blot stated increases in the expressions of collagen I, α-SMA, and P4HA3 in the Ang II group (Figures 1E,F, *P* < 0.05). The above results implicated that Ang II treatment is able to induce atrial fibrosis, in addition to suppressing the expression of miR-205, and elevating the expression of P4HA3.

### Ang II Contributes to Proliferation and Migration of Rat Atrial Fibroblasts

**Figure 2:**
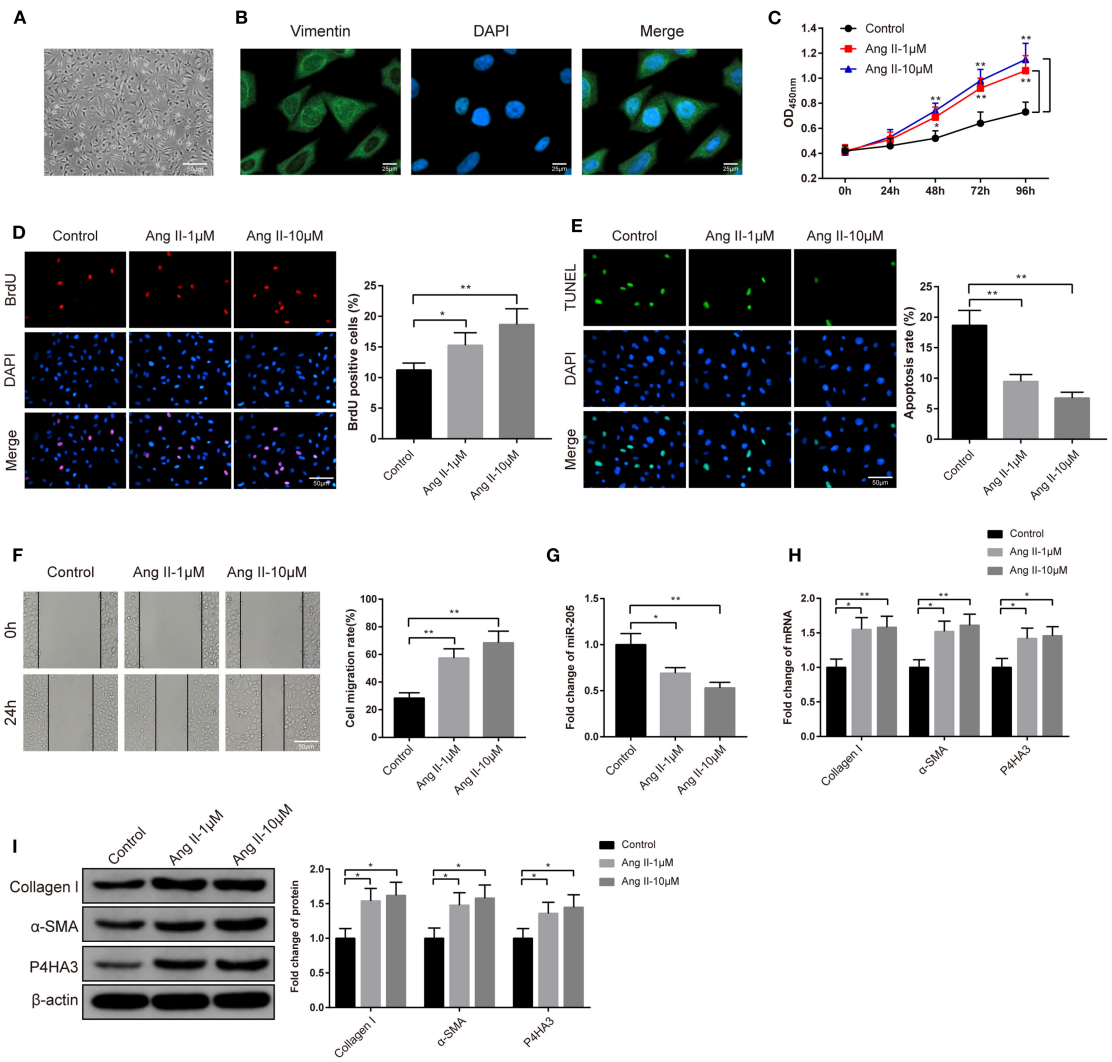
Effect of Ang II on atrial fibrosis. The morphology of rat atrial fibroblasts was observed under an inverted microscope **(A)**. Immunofluorescence fluorescence of Vimentin was utilized to identify atrial fibroblasts **(B)**. CCK-8 assay **(C)** and BrdU assay **(D)** were adopted for measurement of the effect of Ang II on the proliferation ability of rat atrial fibroblasts, TUNEL staining for assessment of the apoptosis rate of atrial fibroblasts **(E)** and cell scratch assay for the performance of migration ability of rat atrial fibroblasts **(F)**. The effect of Ang II on the expression of miR-205 was determined by qRT-PCR **(G)**. The expression of collagen I, α-SMA, and P4HA3 in rat atrial fibroblasts were inspected by qRT-PCR **(H)** and Western blot **(I)**; *P < 0.05, **P < 0.01; Ang II, angiotensin II.

The rat atrial fibroblasts were isolated, cultured, and then observed under an inverted microscope. After 48 h of culture, atrial fibroblasts of rat presented in irregular and spindle shape (Figure 2A). Immunofluorescence analyzed that atrial fibroblasts were positive for Vimentin staining and atrial fibroblasts were successfully isolated (Figure 2B). Subsequently, the atrial fibroblasts were given 1 μM or 10 μM Ang II to determine the optimal concentration of Ang II on atrial fibrosis. The high cell viability in the Ang II group rather than in the Control group was assessed by CCK-8 assay (Figure 2C, *P* < 0.05). Besides, BrdU assay on the proliferation ability of atrial fibroblasts stated that exposure to Ang II increased the number of BrdU-positive cells in atrial fibroblasts (Figure 2D, *P* < 0.05). Analysis of TUNEL staining described that in comparison to the Control group, the Ang II group had suppressed cell apoptosis rate (Figure 2E, *P* < 0.01). Cell scratch assay exhibited that there was increased migration rate in the Ang II group rather than the Control group (Figure 2F, *P* < 0.01). Significant decreased miR-205 expression in the Ang II group was noticed by qRT-PCR detection (Figure 2G, *P* < 0.05). qRT-PCR and Western blot highlighted that the levels of collagen I, α-SMA, and P4HA3 in atrial fibroblasts of Ang II group were notably increased in comparison to the Control group (Figures 2H,I, *P* < 0.05). Taken these data together, Ang II may promote the proliferation and migration of rat atrial fibroblasts, repress miR-205 expression and enhance P4HA3 expression.

### MiR-205 Negatively Targets P4HA3

**Figure 3:**
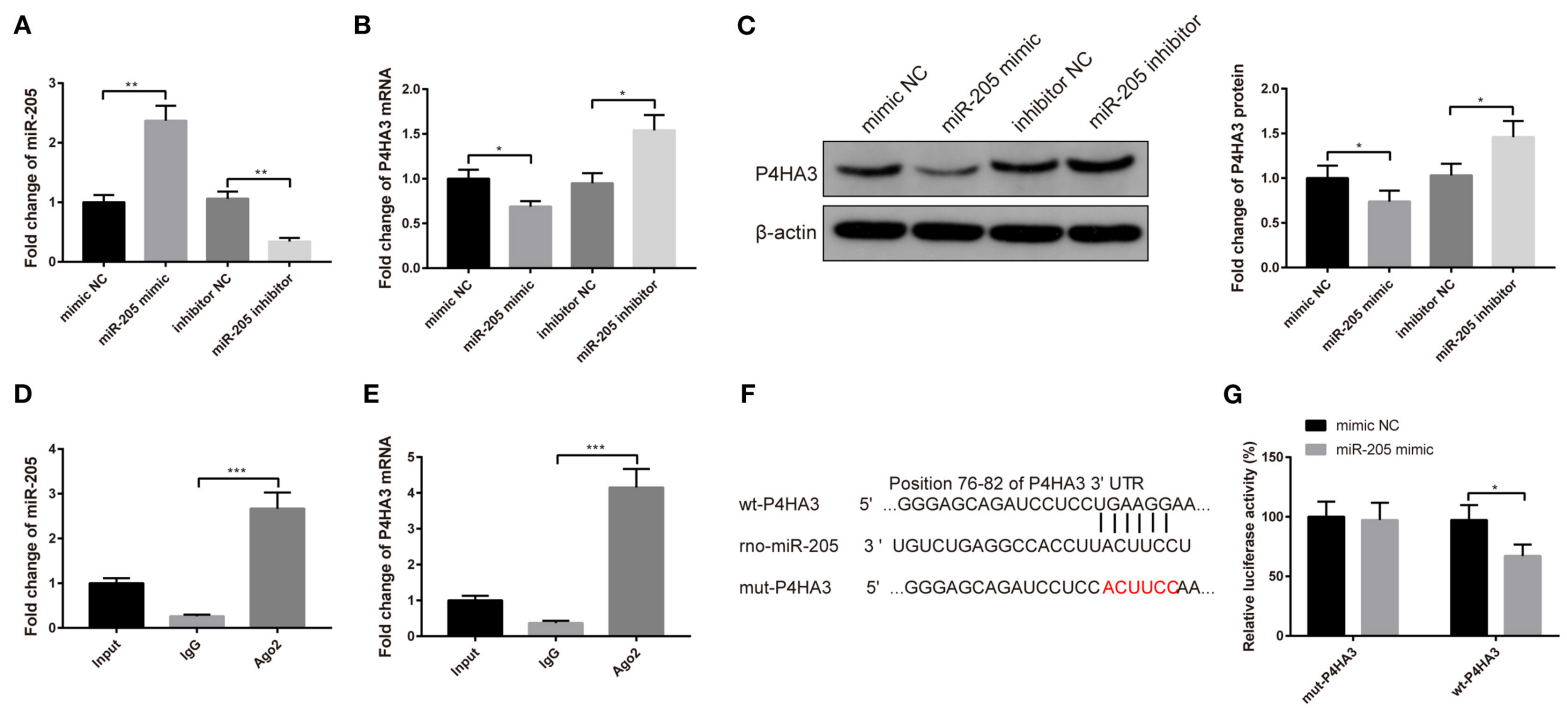
P4HA3 is a target gene of miR-205 in rat atrial fibroblasts. After transfection with miR-205 mimic or miR-205 inhibitor, the mRNA expression of miR-205 was detected by qRT-PCR **(A)**, and the mRNA and protein levels of P4HA3 were measured by qRT-PCR **(B)** and Western blot **(C)**. RIP experiments verified that Ago2 antibody significantly enriched the mRNA of miR-205 and P4HA3 **(D,E)**. The binding site of miR-205 and P4HA3 was predicted by TargetScan, and sequences were designed according to the binding site **(F)**. Dual-luciferase reporter gene assay was applied to verify the binding site of miR-205 and P4HA3 **(G)**; *P < 0.05, **P < 0.01, ***P < 0.001; RIP, RNA immunoprecipitation.

The atrial fibroblasts were transfected with miR-205 mimic or miR-205 inhibitor to explore the relationship between miR-205 and P4HA3. Transfection with miR-205 mimic heightened the mRNA level of miR-205 (Figure 3A, *P* < 0.01) and restrained mRNA and protein expressions of P4HA3 (Figures 3B,C, *P* < 0.05), whereas transfection with miR-205 inhibitor enhanced P4HA3 expression and reduced miR-205 expression (Figures 3A–C, *P* < 0.05).

Additionally, RIP result manifested that in rat atrial fibroblasts, Ago2 antibody significantly enriched mRNA of miR-205 and P4HA3 compared with IgG antibody (Figures 3D,E, *P* < 0.001). The binding sites of miR-205 and P4HA3 by TargetScan are shown in Figure 3F. Results of the dual-luciferase reporter gene assay presented that insertion of the mut2-P4HA3 sequence did not change the luciferase activity of miR-205 mimic, whereas the luciferase activity of miR-205 mimic markedly decreased after insertion of the wt-P4HA3 sequence and mut1-P4HA3 sequence (Figure 3G, *P* < 0.05). Collectively, miR-205 can negatively mediate P4HA3 expression in rat atrial fibroblasts.

### Effect of miR-205/P4HA3 Axis on Proliferation and Migration of Atrial Fibroblasts

**Figure 4:**
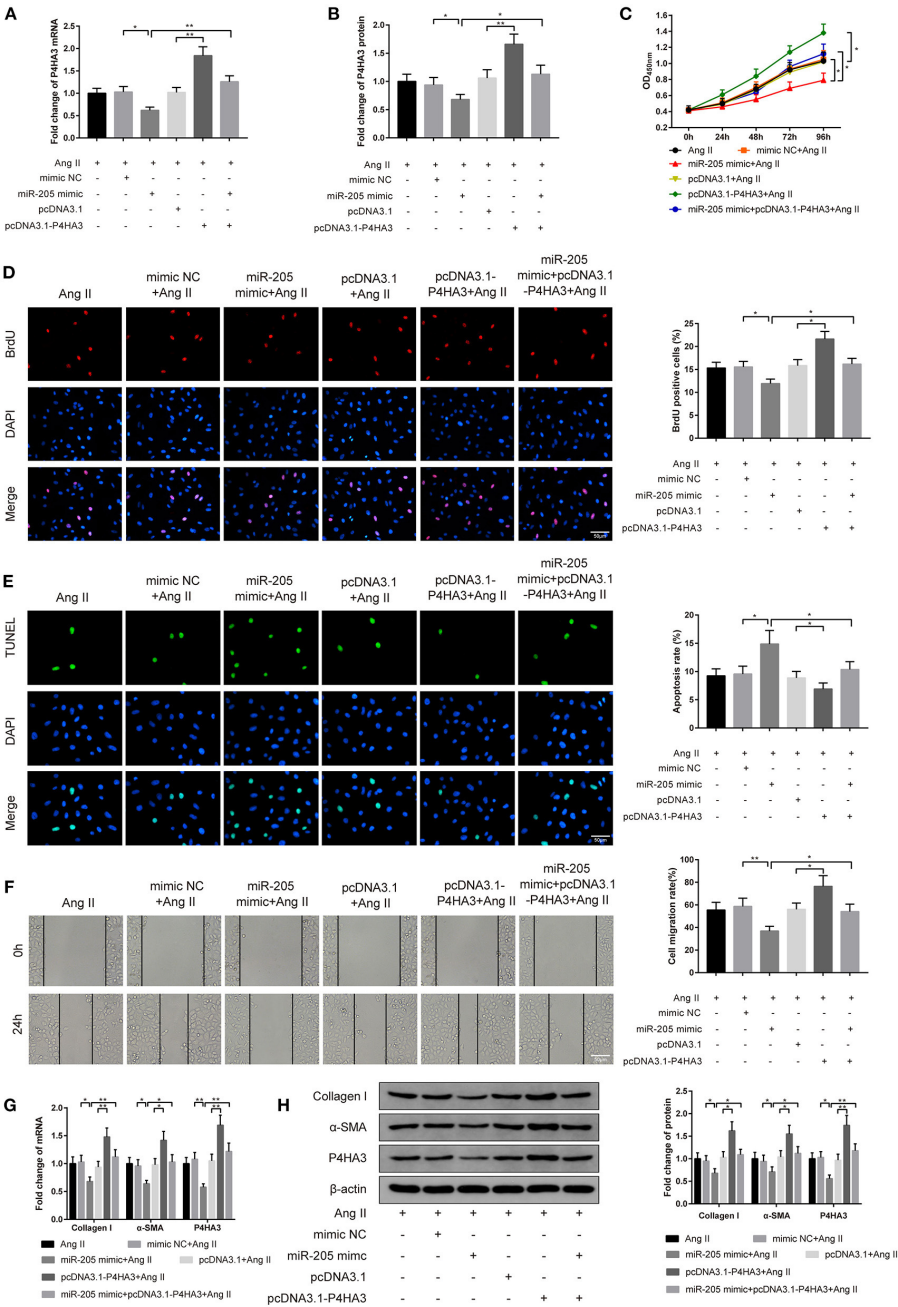
MiR-205 mediates P4HA3 to restrain atrial fibroblast proliferation and migration. The rat atrial fibroblasts were transfected with miR-205 mimic or pcDNA3.1-P4HA3, or cotransfected with miR-205 mimic and pcDNA3.1-P4HA3 followed by exposure to 1 μM Ang II. qRT-PCR **(A)** and Western blot **(B)** were employed for detection of P4HA3 expression, CCK-8 for the performance of the activity of rat atrial fibroblasts **(C)**, BrdU assay for observation of the proliferation ability of rat atrial fibroblasts **(D)**, TUNEL staining for inspection of atrial fibroblast apoptosis **(E)**, and cell scratch assay for evaluation of rat atrial fibroblast migration ability **(F)**. The mRNA and protein expressions of collagen I, α-SMA, and P4HA3 were displayed by qRT-PCR **(G)** and Western blot **(H)**; *P < 0.05, **P < 0.01; Ang II, angiotensin II.

The following study was arranged to explore the role of miR-205/P4HA3 axis in atrial fibroblasts *in vitro*. The rat atrial fibroblasts were transfected with miR-205 mimic or pcDNA3.1-P4HA3, or cotransfected with miR-205 mimic and pcDNA3.1-P4HA3 followed by treating with 1 μM Ang II. Analyses of qRT-PCR and Western blot displayed the rises in P4HA3 expression in the pcDNA3.1-P4HA3+Ang II group (Figures 4A,B, *P* < 0.01, vs. pcDNA3.1+Ang II group) and in the miR-205 mimic+pcDNA3.1-P4HA3+Ang II group (Figures 4A,B, *P* < 0.05, vs. miR-205 mimic+Ang II), in addition to the decrease in P4HA3 level in the miR-205 mimic+Ang II group (Figures 4A,B, *P* < 0.05, vs. mimic NC+Ang II group).

CCK-8 assay on cell viability manifested that the co-effect of miR-205 overexpression and Ang II lowered the activity of atrial fibroblasts in comparison to the cotreatment of mimic NC and Ang II on cell proliferation ability. Besides, there was enhanced viability of atrial fibroblasts in the pcDNA3.1-P4HA3+Ang II group rather than the pcDNA3.1+Ang II group. Compared with the miR-205 mimic+Ang II group, the miR-205 mimic+pcDNA3.1-P4HA3+Ang II group had higher atrial fibroblast viability (Figure 4C, *P* < 0.05).

The BrdU assay corroborated the results of CCK-8 assay that the number of BrdU-positive cells was significantly suppressed in the miR-205 mimic+Ang II group when compared with the mimic NC+Ang II group, whereas in comparison to the pcDNA3.1+Ang II group, the number of BrdU-positive cells was obviously elevated in the pcDNA3.1-P4HA3+Ang II group (Figure 4D, *P* < 0.05).

The results of TUNEL staining stated that the apoptotic rate was increased after atrial fibroblasts were transfected with miR-205 mimic and subjected to Ang II (Figure 4E, *P* < 0.05, vs. mimic NC+Ang II group), and the apoptosis rate was decreased after cells were transfected with pcDNA3.1-P4HA3 and exposed to Ang II (Figure 4E, *P* < 0.05, vs. pcDNA3.1+Ang II group). Different expression patterns was found by cell scratch assay (Figure 4F, *P* < 0.05).

qRT-PCR and Western blot presented that there were declined expressions of collagen I, α-SMA, and P4HA3 in the miR-205 mimic+Ang II group (Figures 4G,H, *P* < 0.05, vs. mimic NC+Ang II group) and elevated levels of collagen I, α-SMA, and P4HA3 in the pcDNA3.1-P4HA3+Ang II group (Figures 4G,H, *P* < 0.05, vs. pcDNA3.1+Ang II group). In summary, overexpression of miR-205 inhibits Ang II-induced fibrosis of rat atrial fibroblasts, while overexpression of P4HA3 can abolish the effect of miR-205 in Ang II-induced atrial fibroblasts.

### MiR-205/P4HA3 Confers Crucial Role in JNK Signaling Pathway

**Figure 5:**
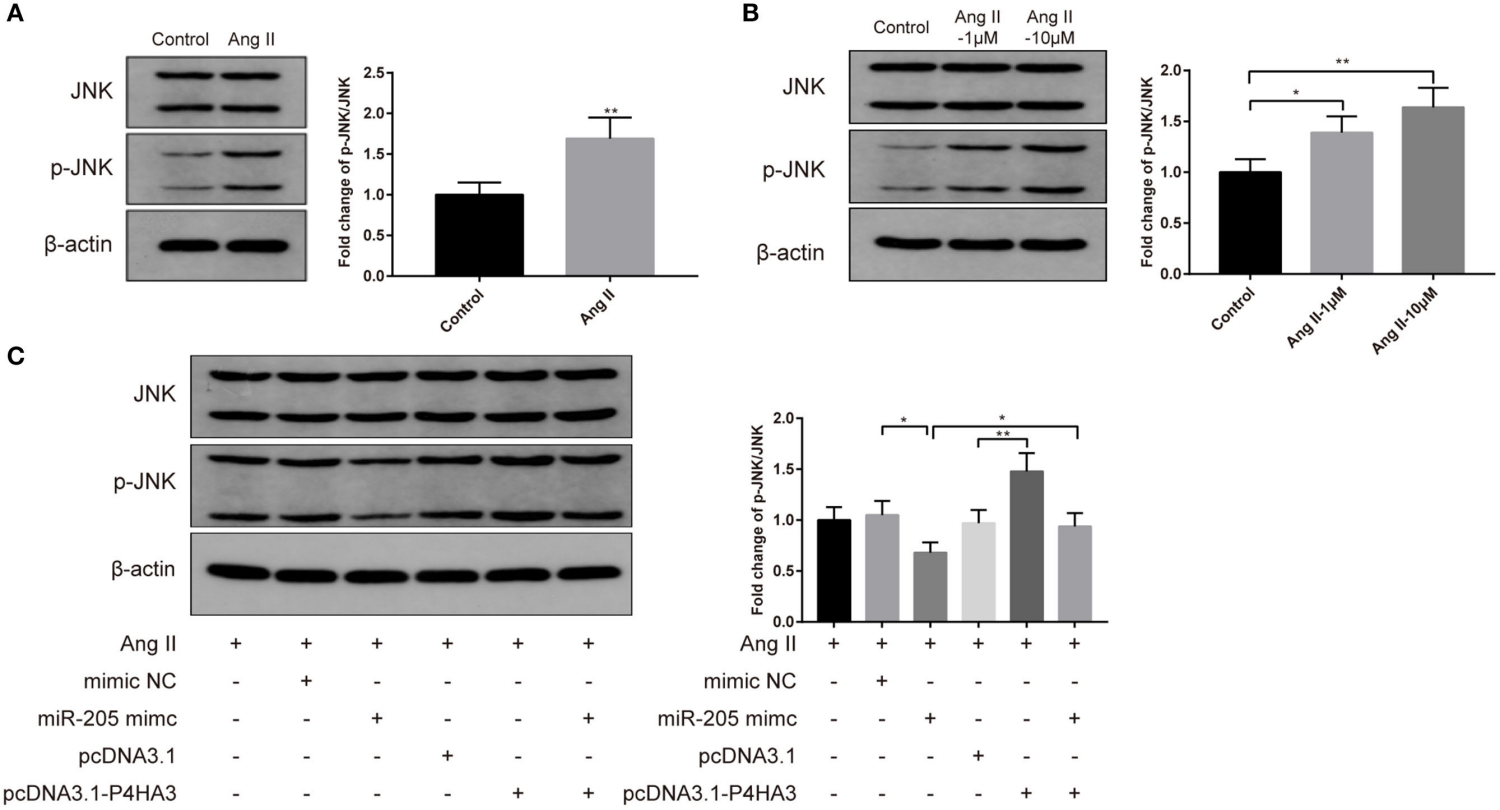
MiR-205 restrains atrial fibrosis through P4HA3 to block JNK pathway. After Ang II injection in rats, the p-JNK expression in atrial tissues was measured by Western blot **(A)**. Western blot was applied to detect the p-JNK expression in Ang II-treated atrial fibroblasts **(B)**. Then, Western blot analysis of p-JNK expression were performed on rat atrial fibroblasts which were transfected with miR-205 mimic, pcDNA3.1-P4HA3, or cotransfected with miR-205 mimic and pcDNA3.1-P4HA3 prior to treatment with Ang II (1 μM) **(C)**; *P < 0.05, **P < 0.01; Ang II, angiotensin II.

To investigate the specific pathway involved in the atrial fibrosis, we characterized the JNK signaling. Results of Western blot displayed that injection of Ang II into rats heightened protein level of p-JNK *in vivo* (Figure 5A, *P* < 0.01), and exposure to Ang II enhanced p-JNK expression in atrial fibroblasts *in vitro* (Figure 5B, *P* < 0.05). Following transfection with pcDNA3.1-P4HA3 further elevated p-JNK expression in Ang II-treated atrial fibroblast (Figure 5C, *P* < 0.01, vs. pcDNA3.1+Ang II group), while transfection with miR-205 mimic reduced the protein level of p-JNK in Ang II-treated atrial fibroblast (Figure 5C, *P* < 0.05, vs. mimic NC+Ang II group), and cotransfection with miR-205 mimic and pcDNA3.1-P4HA3 increased p-JNK level (Figure 5C, *P* < 0.05, vs. miR-205 mimic+Ang II group). The above results suggested that the JNK pathway can be activated during atrial fibrosis, while the miR-205/P4HA3 axis can inhibit the activation of the JNK pathway.

### Effect of JNK Signaling Pathway on Atrial Fibrosis

**Figure 6:**
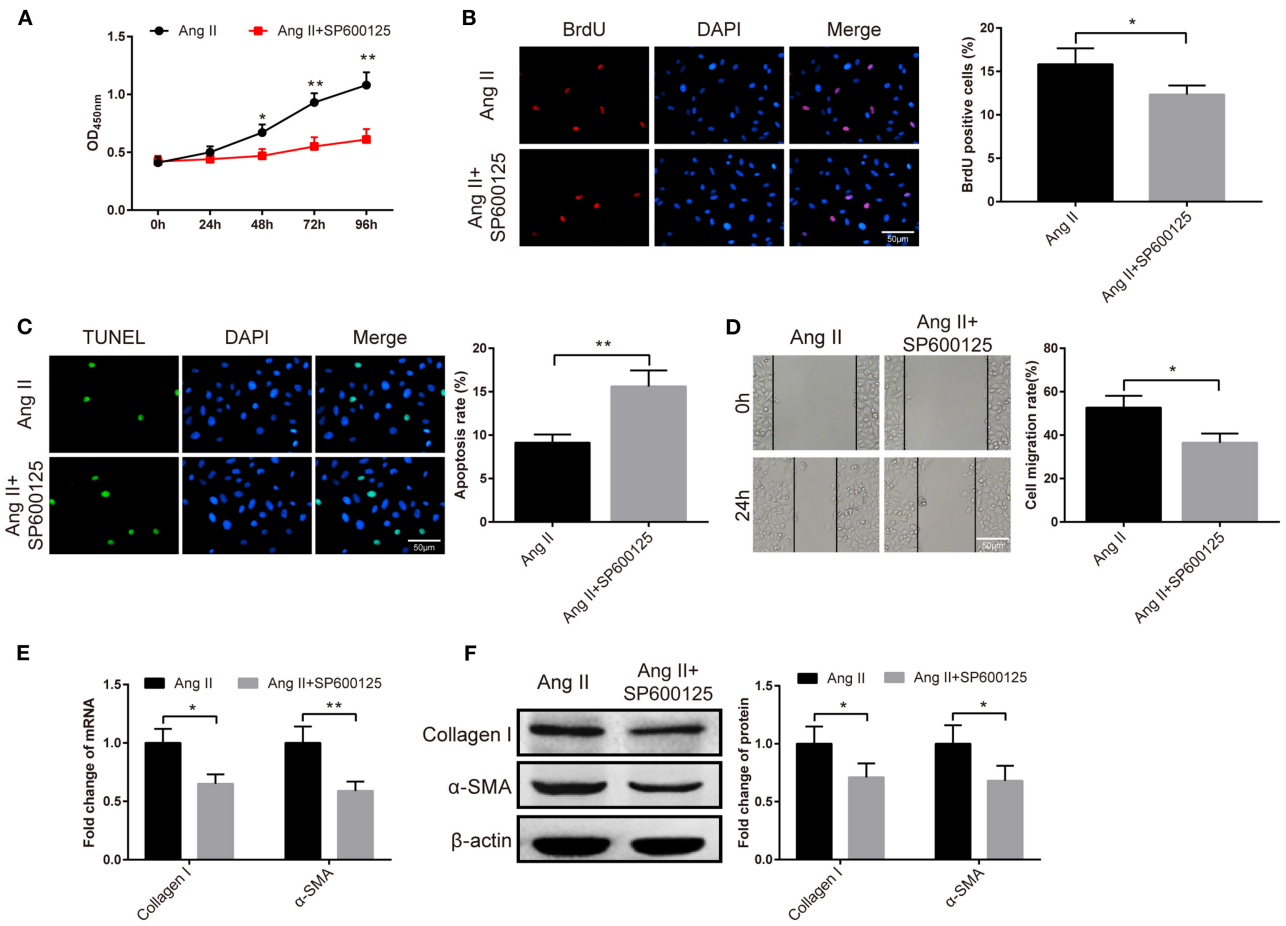
The JNK pathway is implicated in the atrial fibrosis. After the fibroblasts were treated with JNK inhibitor SP600125, the cell viability was measured by CCK-8 assay **(A)**, the proliferation ability was detected by BrdU assay **(B)**, cell apoptosis was evaluated by TUNEL staining **(C)**, and cell migration ability was examined by cell scratch assay **(D)**. Then, qRT-PCR and Western blot were used to assess the mRNA and protein expressions of collagen I and α-SMA **(E,F)**; *P < 0.05, **P < 0.01.

The fibroblasts were given JNK inhibitor SP600125 to probe the role of the JNK signaling pathway in atrial fibrosis. The results of CCK-8 manifested the pronounced fall in the viability of fibroblasts in the Ang II+SP600125 group than the Ang II group (Figure 6A, *P* < 0.05). Subsequently, BrdU assay, TUNEL staining and cell scratch assay demonstrated that cells in the Ang II+SP600125 group possessed higher apoptotic rate (Figure 6C, *P* < 0.01), fewer BrdU-positive cells (Figure 6B, *P* < 0.05) and lower migration rate (Figure 6D, *P* < 0.05). Analyses of qRT-PCR and Western blot revealed that the expression levels of collagen I and α-SMA in atrial fibroblasts of the Ang II+SP600125 group were obviously lower than those in the Ang II (Figures 6E,F, *P* < 0.05). The above data indicated that suppression of the JNK signaling pathway may inhibit Ang II-induced migration and proliferation of fibroblasts.

## Discussion

Atrial fibrosis is a common feature of clinical AF (20). Atrial fibroblasts are the main effector cells, and the migration and proliferation of atrial fibroblasts are central in AF-promoting structure remodeling (21, 22). Herein, we characterized atrial fibrosis in cell and animal models, observed changes in the abilities of atrial fibroblast migration and proliferation, along with miR-205 and its target gene P4HA3, in atrial fibrosis caused by Ang II, and determined whether miR-205 overexpression attenuates atrial fibrosis and AF in models. We found that miR-205 repressed proliferation and migration of rat fibroblasts through P4HA3 suppression and JNK signaling pathway blockage to ameliorate atrial fibrosis.

It is also likely that miRNAs interfere with processes underlying cardiovascular diseases including structural remodeling and cardiac electrical remodeling (23). However, there remains a paucity of evidence surrounding miR-205 in AF. Recently, Wei Tao et al. identified that miR-205-5p contributes to pulmonary vascular smooth muscle cell proliferation through targeting Erk1/2 in pulmonary arterial hypertension (24). Xue et al. also illustrated that in psoriatic mice, miR-205-5p may inhibit keratinocyte hyperproliferation and excessive angiogenesis via deactivating the Wnt/b-catenin and MAPK pathways (25). In light of recent data, we hypothesized that miR-205 may be a candidate to participate in AF-promoting atrial fibrosis. To test this possibility, we first evaluated the expression of miR-205 in AF models. We found lowly expressed miR-205 in the rat atrial fibroblast model and atrial tissue model which were constructed by Ang II infusion. To further explore the role of miR-205 in AF progression, the gain-of-function method was exploited. Overexpression of miR-205 restrained atrial fibroblast proliferation and migration, as evidenced by elevated apoptosis rate of atrial fibroblasts, declined cell viability, suppressed cell migration capacity, and repressed expressions of fibrotic markers in atrial fibroblasts. Collectively, miR-205 participated in atrial fibrosis progression through suppression of atrial fibroblast migration and proliferation. Notably, P4HA3 was noticed as a downstream effector of miR-205. Hence, we gave a conjecture that P4HA3 would provide a clear window into atrial fibrosis after Ang II stimulation. P4H catalyzes the formation of 4-hydroxyproline, which is essential for the proper three-dimensional folding of newly synthesized procollagen chains (26). P4HA3 is one of the three P4HA isoforms that has been detected with a notably elevated expression in gastric cancer and is correlated with a poor prognosis in breast cancer (27). Under this setting, we sought to explore the hypothesis by profiling the expression of P4HA3 in atrial tissues. The enhanced P4HA3 expression occurred with Ang II stimulation. Then, P4HA3 was discovered to facilitate Ang II-induced atrial fibrosis, as evidenced by enhanced cell viability, elevated cell migration capacity, increased expressions of fibrotic markers, and inhibited apoptosis rate of atrial fibroblasts after cotreatment of pcDNA3.1-P4HA3 and Ang II. In line with these current observations, a few recent studies also have shown similar findings. Downregulation of P4HA1 is involved in liraglutide-mitigated myocardial fibrosis through the CD36-JNK-AP1 pathway (28). In vascular smooth muscle cells, miR-124-3p presents itself in the repression of collagen synthesis in atherosclerotic plaques by targeting P4HA1 (29). Additionally, P4HA3 was proven to be a target gene of miR-205 by dual-luciferase reporter gene assay and RIP experiment. The gain-of-function method for functional miR-205/P4HA3 unfolded that miR-205 upregulation inhibits Ang II-induced atrial fibrosis, while overexpression of P4HA3 reversed the coeffect of miR-205 upregulation and Ang II. The data indicate that miR-205 mitigates atrial fibrosis through negatively mediating P4HA3 to inhibit migration and proliferation of atrial fibroblasts.

As members of the MAPK family, the JNKs regulate eukaryotic cell responses to a wide range of abiotic and biotic stress insults (30). The Rac1/JNK signaling participates in malignant transformation of fibrosis (31). Furthermore, JNK signaling plays an integral role in several crucial mechanisms operating in renal fibrosis (32). With regard to the functional JNK pathway in fibrosis, the implication of the JNK pathway in Ang II-induced atrial fibrosis was investigated. We proposed that Ang II treatment intensified atrial fibrosis and activation of the JNK pathway, while miR-205 blocked this signaling.

In conclusion, the present study clarifies the critical role of the miR-205/P4HA3 axis in the course of atrial fibrosis, which is mediated by the inhibition of proliferation and migration abilities of Ang II-induced atrial fibroblasts. Notably, our findings imply that the blockage of the JNK pathway interfered with miR-205/P4HA3-dependent actions is postulated to be a reasonable approach for atrial fibrosis and AF. Our results suggest that miR-205 may serve as a novel therapeutic or prophylactic target in AF.

## Data Availability Statement

The raw data supporting the conclusions of this article will be made available by the authors, without undue reservation.

## Ethics Statement

The animal study was reviewed and approved by Nanfang Hospital, Southern Medical University.

## Author Contributions

SYZ, ZZX, and DPKR conceived the ideas. SYZ, ZZX, and DPKR designed the experiments. CQX and XML performed the experiments. XC and XL analyzed the data. JLL and XL provided critical materials. ZZX and DPKR wrote the manuscript. SYZ supervised the study. ZZX and DPKR contributed equally to this research. All the authors have read and approved the final version for publication.

## Conflict of Interest

The authors declare that the research was conducted in the absence of any commercial or financial relationships that could be construed as a potential conflict of interest.
